# Supplementary material for: Modulatory dynamics of periodic and aperiodic activity in respiration-brain coupling
Source: Nat Commun. 2023 Aug 5;14:4699. doi: 10.1038/s41467-023-40250-9 (PMC10404236; doi:10.1038/s41467-023-40250-9)
Supplement: Supplementary file 1 — Supplementary information [file 41467_2023_40250_MOESM1_ESM.pdf]

Modulatory dynamics of periodic and aperiodic activity in respiration-brain coupling

Supplementary Information

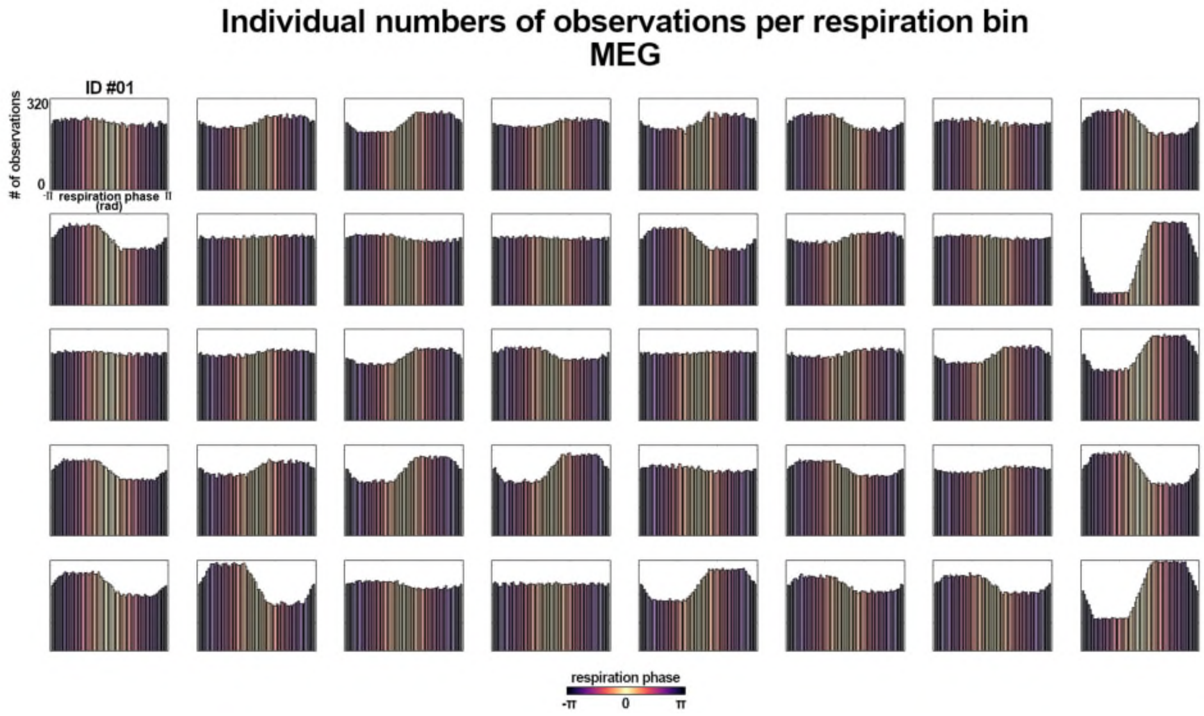

Fig. S1. Individual numbers of observations per respiration phase bin in the MEG sample (N = 40). Continuous respiration phase was partitioned into k = 60 phase bins (x-axis) to determine the number of 1/f slope estimations (yielded by the SPRiNT algorithm) per respiration phase (y-axis). For details on respiration phase-locked slope estimations, see Fig. 1 and Methods. Respiration phase is colour-coded in accordance with main figures.

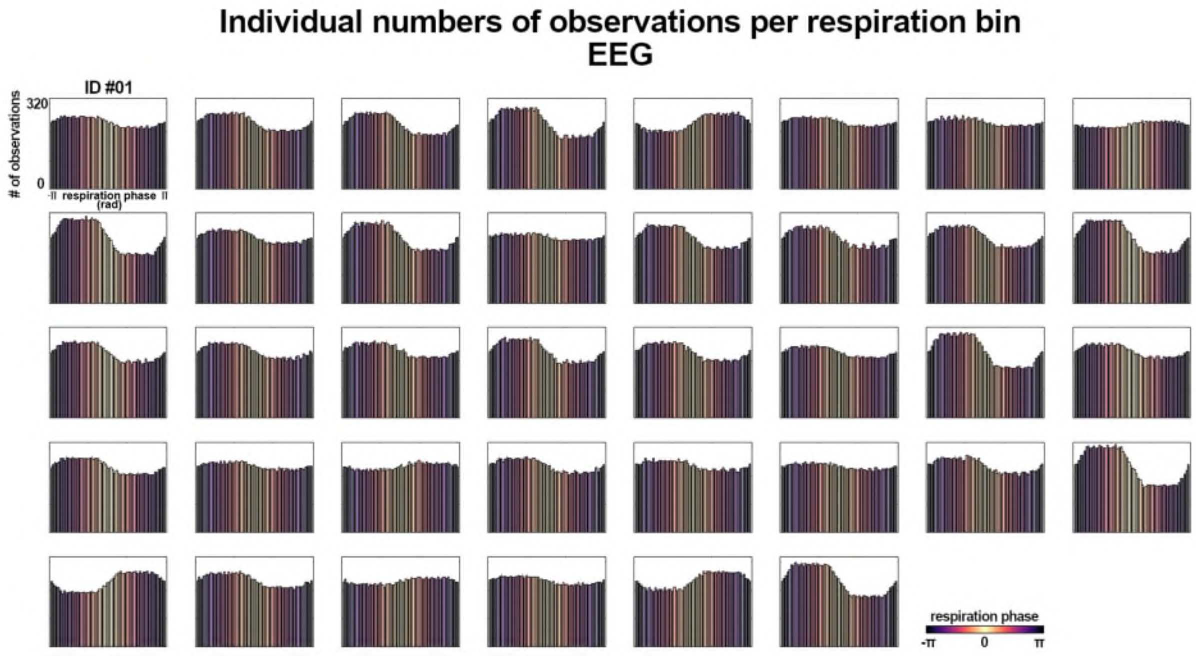

Fig. S2. Individual numbers of observations per respiration phase bin in the EEG sample (N = 38). Continuous respiration phase was partitioned into k = 60 phase bins (x-axis) to determine the number of 1/f slope estimations (yielded by the SPRiNT algorithm) per respiration phase (y-axis). For details on respiration phase-locked slope estimations, please Fig. 1 and Methods. Respiration phase is colour-coded in accordance with main figures.

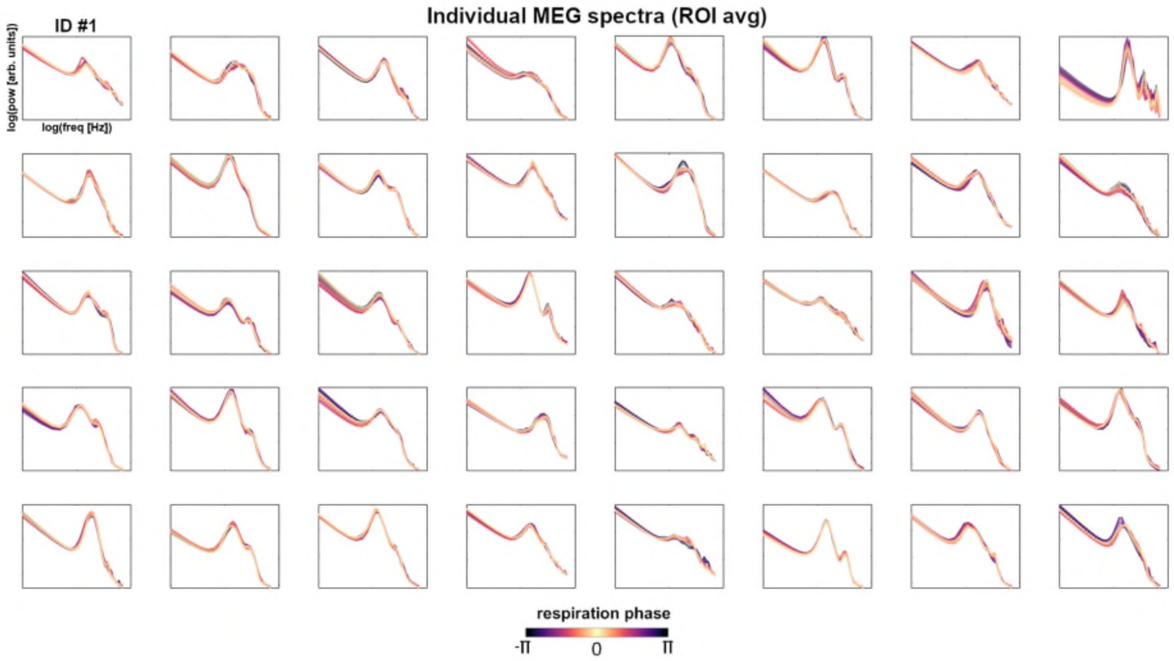

Fig. S3. Individual MEG power spectra (averaged across sensors within the parieto-occipital ROI) in log-log transform. For each participant, we show power spectra in the frequency range from 1 Hz to 40 Hz for each of the 60 respiratory phase bins (colour-coded).

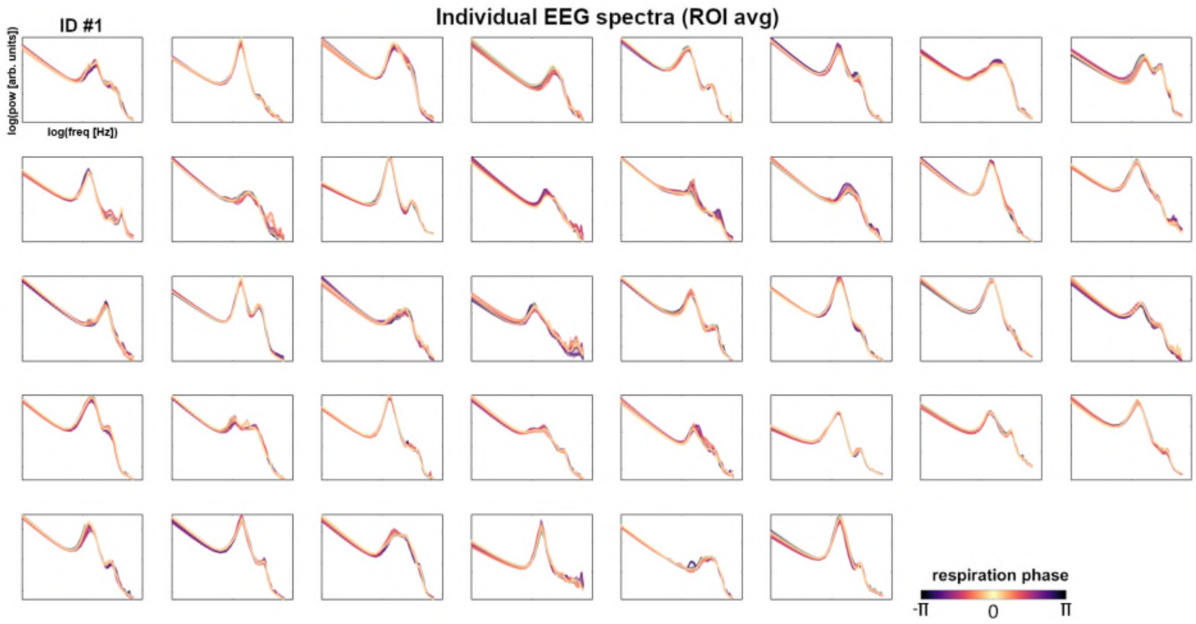

Fig. S4. Individual EEG power spectra (averaged across channels within the parieto-occipital ROI) in log-log transform. For each participant, we show power spectra for each of the 60 respiratory phase bins (colour-coded).
